# Development of Antibacterial Protective Coatings Active against MSSA and MRSA on Biodegradable Polymers

**DOI:** 10.3390/polym13040659

**Published:** 2021-02-23

**Authors:** Iva Rezić, Mislav Majdak, Vanja Ljoljić Bilić, Ivan Pokrovac, Lela Martinaga, Maja Somogyi Škoc, Ivan Kosalec

**Affiliations:** 1 Department of Applied Chemistry, Faculty of Textile Technology, University of Zagreb, 10000 Zagreb, Croatia; mmajdak@ttf.hr (M.M.); lela.martinaga@ttf.hr (L.M.); 2 Faculty of Pharmacy and Biochemistry, Institute for Microbiology University of Zagreb, 10000 Zagreb, Croatia; vljoljic@pharma.hr (V.L.B.); ivan.pokrovac.fbf@gmail.com (I.P.); 3 Department of Material Testing, Faculty of Textile Technology, University of Zagreb, 10000 Zagreb, Croatia; maja.somogyi@ttf.hr

**Keywords:** antibacterial coating, *Staphylococcus aureus*, MRSA, nanoparticles, 3D printing

## Abstract

In this work the in vitro antimicrobial activity of colloidal solutions of nine different commercially available nanoparticles were investigated against *Staphylococcus aureus* strains, both methicillin-sensitive (MSSA) and methicillin-resistant (MRSA). Research covered antimicrobial investigation of different metal and metal-oxide nanoparticles, including Ag 10 nm, Ag 40 nm, Al_2_O_3_ 100 nm, Au 20 nm, Pt 4 nm, TiO_2_ 100 nm, Y_2_O_3_ 100 nm, ZnO 100 nm and ZrO_2_ 100 nm nanoparticles. Such materials were foreseen to be applied as coatings on 3D-printed biodegradable polymers: i.e., catheters, disposable materials, hospital bedding items, disposable antimicrobial linings and bandages for chronic wounds. Therefore, the antimicrobial activity of the nanoparticles was determined by agar well diffusion assays and serial microdilution broth assays. In addition, the chromatographic characterization of elements present in trace amounts was performed as a method for tracing the nanoparticles. Moreover, the potential of preparing the rough surface of biodegradable polymers for coating with antimicrobial nanoparticles was tested by 3D-printing fused deposition methodology. The in vitro results have shown that particular nanoparticles provided powerful antimicrobial effects against MSSA and MRSA strains, and can be easily applied on different biopolymers.

## 1. Introduction

By the year 2050, more people could die from the infections caused by antibiotic-resistant bacteria than from AIDS, tuberculosis and viral hepatitis combined together. Only in Europe 25,000 deaths per year and costs of over €1.5 billion are the consequences of the activity of resistant microorganisms. Moreover, the estimations show that in the next 30 years more people could die from the infections caused by antibiotic-resistant bacteria than from cancer [1]. Despite the reductions in the incidence of infections since 2005, *Staphylococcus aureus* infections still account for a significant amount of morbidity and mortality in the United States [2]. Antimicrobially-active biodegradable polymers that contain metal and metal oxide nanoparticles present in the outer layer of coatings can have important applications in medicine and medical materials. Therefore, such materials might have potential usage against methicillin-resistant *S. aureus* (MRSA). Moreover, polymer materials that are not produced by classical fiber production and spinning technology, but by other processes (such as additive technology or 3D printing) can easily be functionalized with metal nanoparticles with the goal to obtain antimicrobial properties. Such materials can be used as catheters, consumables, dressings and wound dressings. In addition, would be possible to apply them in the production of protective masks and protective suits and to answer to the most prominent problems of today’s medical materials [3].

Therefore, this research work was focused on solutions against MSSA and MRSA microorganisms, known members of the “*superbugs*” group. Those microorganism strains are resistant to a wide range of antibiotic-based penicillin drugs (e.g., methicillin, dicloxacillin, nafcillin and oxacillin) and belong to the *Staphylococcaceae* family. *Staphylococcus aureus* is a Gram-positive bacteria that has spherical form, and that is today one of the most significant pathogens in the world. The most problematic issue related to the “*superbugs*” problem, is the percentage of deadly outcomes in hospitals, which ranges from 10–30% [4].

The extraordinary antimicrobial activity of nanoparticles is the consequence of their dimensions that are in the range of one to one hundred nanometers. When compared to the dimension of microorganisms, it can be clearly seen that nanoparticles are far smaller than the microorganisms, including fungi, Gram-positive and Gram-negative bacteria. Moreover, nanoparticles made of metals and metal oxides can be toxic or non-toxic to human cells. Padmavathy and Vijayaraghavan have investigated this topic and proved that nanoparticles are biocompatible with human cells [5], which makes them suitable additives for polymers, textiles and surfaces that come into direct contact to the human body. Therefore, there is a high research interest and strong focus on the interaction mechanisms between nanoparticles (NPs) and microorganisms.

Current results show that nanoparticles of metals and metal oxides influence microorganisms in both ways: at their surfaces and penetrating through the microorganisms cells. Although the mechanisms of the interaction of metal oxide nanoparticles with MRSA strains are still under investigation, different nanoparticles have shown antimicrobial activity on MRSA, but also on different other microorganisms, including bacteria and fungi. Interesting work from Pandey et al. [6] has shown that the method of stabilization of nanoparticles strongly influences the antimicrobial properties. In this research, milk-stabilized silver nanoparticles (AgNPs) were found to be more toxic to bacteria than to fungi. Model microorganisms investigated were following: Gram-positive microorganisms *Bacillus subtilis* and *Staphylococcus aureus*, Gram-negative microorganisms *Salmonella typhi* and *Escherichia coli*, and fungi *Aspergillus fumigatus*, *Aspergillus ochraceus* and *Penicillium chrysogenum* [6]. Moreover, sodium alginate-stabilized silver/mesoporous silica nanohybrids demonstrated excellent antibacterial activity against both Gram negative and Gram positive bacteria [7]. Thus, stabilization of nanoparticles has potential to be used for various antibacterial applications [8,9,10,11].

Some of the most prominent applications of antimicrobial nanoparticles are foreseen as sophisticated medical materials. 3D printing is one of the most efficient methodologies for producing biodegradable polymers in different shape and size which are compatible with the human organism. Rastin et al. have used a three-dimensional bioprinting approach with the goal to construct complex structures suitable for tissue regeneration [12]. In addition, Peak et al. designed a hydrolytically degradable polymer and loaded it with a nanoengineered bioink with therapeutic proteins to direct cell function in a 3D printed construct. The bioink has been developed from polymer and two dimensional synthetic nanoparticles [13]. The authors have shown that by changing the process parameters (mechanical properties, swelling kinetics, and degradation rate of 3D printed constructs) the concentration of nanosilicates varies, and enables the sustained release of therapeutics from 3D printed structures. This approach is foreseen to be used in engineering of 3D complex tissue structures for regenerative medicine [13].

In such medical applications, the crucial parameters are the mechanisms of the antimicrobial effects of nanoparticles. Recent investigations show that the antibacterial activities of NPs depend on two main factors: (i) the physicochemical properties of the NPs and (ii) the type of bacteria [14]. Mohammad et al. have shown that nanoparticles are able to attach to the membrane of bacteria by electrostatic interactions and disrupt the integrity of the bacterial membrane [14]. The step is triggered by the induction of oxidative stress which occurs after the formation of free radicals. In the next step, the administration and penetration of NPs to bacteria cell occurs. Therefore, the toxicity of copper NPs (CuNPs) depended on the combination of different process parameters such are: the temperature, aeration and pH value, as well as the concentration of NPs and concentration of bacteria. The parameters that were increasing the toxicity of the nanoparticles were: high temperature ranges and high aeration, and low pH due to the decrease in the agglomeration of NPs. Moreover, the lower agglomeration provided the surface area that was more available for interaction with bacterial membranes and solubilization of metal ions. In conclusion, such conditions were favorable for leading higher toxicity ranges of nanoparticles to model microorganisms [14,15].

The mechanism of silver nanoparticles’ (AgNPs) penetration through the cell wall of Gram-negative bacteria described by Varner in a U.S. EPA report [16], followed several steps: the structural change in the cell membrane increases the cell permeability, leading to an uncontrolled transport of nanoparticles through the cytoplasmic membrane, and ultimately cell death. The proposed process is related to the formation of free radicals and subsequent free radical-induced membrane damage. After the membrane damaging, the ions move into the cells and lead to the production of reactive oxygen species. Then, due to the membrane damage caused by the nanoparticles, the cells cannot effectively extrude the metal ions and limit their effect [16].

The toxicity of different nanoparticles depends on their chemistry: e.g., among NPs such as CuO, NiO, ZnO, and Sb_2_O_3_ used against *E. coli, B. subtilis,* and *S. aureus*, CuO NPs have the highest toxicity, followed by ZnO (except for *S. aureus*), NiO and Sb_2_O_3_ NPs. [14]. In addition to the chemical composition, the size and concentration [15], also the shape of nanoparticles induces their antimicrobial activity: strong antibacterial activity of triangular AgNPs against the Gram-negative bacteria was in correlation to their large surface area to volume ratios and their crystallographic surface structures [16]. The shape of the nanoparticles can be combined with the shape and the chemical composition of the 3D printed polymer bioconstructions produced by bioprinting. Bioprinting is therefore an emerging powerful fabrication method, which enables the rapid assembly of 3D bioconstructs with dispensing cell-laden bioinks in predesigned locations. In addition, it can be promising tool in biomedical applications, which can exploit the advantage of conductive polymers in the 3D bioprinting technology [17]. Therefore, the hydrogel bioinks and their 3D bioprinting with high electrical conductivity, biocompatibility and degradability can synergize some new applications for tissue and neural engineering [18]. Moreover, by using nanoparticles in antimicrobial coatings on 3D printed biodegradable polymers, we can make disposable and eco-friendly materials which will not present the threat to the environment and to organisms in it.

Products made from biodegradable polymers have biodegradability and composting properties. Their usage is growing annually by 10% to 20%, and one of the most common materials among biodegradable polymers is polylactide (PLA). It is a polymer obtained from natural raw materials, such as corn and potatoes. Its monomer is lactic acid, and homopolymers are formed by polymerization in two steps: polycondensation of lactic acid, and opening of the lactide ring. PLA, like other aliphatic polyesters (such as poly ε-caprolactone and polyhydroxybutyrate) is biodegradable in the human body and in nature, but due to the high crystallinity and molecular weight of polymer molecules, its biodegradability is slow.

The use of PLA for medical purposes dates back to the early 80′s of the last century, as a good replacement for metal implants, such as rods, screws and scaffolds, which are used to fix bones, and to help heal damaged tissues. Thanks to biodegradation, PLA rods and screws do not need to be removed by surgery like conventional metal aids, and show good compatibility with the human body, so it is used as a surgical suture, and for skin grafts.

Additive technology, also known as 3D printing, is the method most commonly used to quickly create the desired dimensions and geometry. Design is performed using a CAD modeling program or computer scanning. After the design process, it is necessary to translate the resulting file into STL format and to use the printer software to set the printing conditions or further design so that the process itself can be performed. After printing, the 3D object is removed from the work surface and undergoes a finish, such as functionalization with antimicrobial coatings. Such materials can be easily modified with antimicrobial active coating by further dip-coating process [19].

Antimicrobial modification of polymer materials by nanoparticles can be enhanced in an ultrasonic field, as was shown by Abramova et al. [19]. The researchers coated textiles with titanium and zinc oxide nanoparticles. Their goal was to obtain antibacterial coatings on textile materials [19], and the results obtained have shown that the modified materials could reduce the number of investigated microorganisms (*Escherichia coli)* by more than 99.99%. Another similar study was performed by Akhavan and Montazer. They performed simultaneous loading of nanoparticles from TiO_2_ on cotton using sonochemistry [20]. By this, they were able to produce self-cleaning materials with UV-protection properties. Sonochemistry influences the mechanical properties of nanocomposite films to a great extent [21,22]. It has been found that sonochemical irradiation improves the mechanical properties of nanocomposite films, and makes the synthesis of nanoparticles easier, faster and possible at lower temperatures [23]. The functionalized polymers have antimicrobial protection against model microorganisms. In this work the most prominent model microorganisms were Gram-positive bacteria (*S. aureus*), Gram-negative bacteria (*E. coli*) and fungi (*Candida albicans*). The inhibition zones of all microorganisms were in range of 3.0 to 3.3 mm [23]. In work that followed these preliminary results applications of the thorn-like nanoparticles were described. Since the size and shape of nanoparticles influence the antibacterial effect these thorn-like species show very efficient results [24,25]. Therefore it can be concluded that the toxicity and antibacterial effects of nanomaterials depend on different input process variables which can easily be controled, such are the chemical and morphological composition of nanoparticles, their size and their shape.

Unfortunately, some of the shapes are toxic to daphnids and other organisms [26]. If the size of nanoparticles that is toxic is compared to others, it can be seen that with smaller diameters (nanoparticles of twenty to thirty nm) induce stronger toxically effects than nanoparticles which have larger dimensions. The is obvious due to the property of smaller nanoparticles which have higher statistical probability to enter the cell of the investigated organism [27]. Cellulose materials can be functionalized with metal nanoparticles through a dip coating process which results in a flexible homogenous and unified coating with antimicrobial efficiency [28,29,30,31,32,33]. The characterization of metal nanoparticles needs to be performed with a methodology which offers precise and accurate analysis of trace amounts of analyte in samples, such is spectroscopy or chromatography. Chromatography is a method which enables simultaneous separation, identification and quantification of samples. It can be performed in columns or on thin layers substrates. In many procedures, the chromatography performed on thin layer of different sorbents is recommended as being a fast and simple approach. By varying the stationary and mobile phases it is possible to develop specially designed methods for monitoring and evaluation of particular components. In addition to ion chromatography, which is mainly applied in the analysis of metals, thin layer chromatography enables efficient evaluation of metals in the form of nanoparticles or nanolayers [34,35,36,37,38,39].

This work was therefore focused on the development of antimicrobial metal and metal oxide nanoparticle formulations on biodegradable polymers. The novelty of this work is therefore in the interdisciplinary approach to design biodegradable polymer carriers as well as their antibacterial coatings with nanoparticles active against MSSA and MRSA microorganisms. The main hypotheses of our work are following: (i) 3D printing enables the design and production of biodegradable polymer with desired shapes and sizes which can act as an antibacterial coating carrier; (ii) the printed carrier that can be easily functionalized by sol-gel methodology; (iii) NPs in the coatings of metals and metal oxides have antibacterial effects against microorganisms resistant to antibiotics, such are MRSA and MSSA; and (iv) characterization of newly produced materials can be performed by using combination of chromatographic, spectroscopic and microscopic techniques. This research follows our previous research work and recent literature findings [31,32,33,34,35,36,37,38,39,40,41,42,43,44,45,46,47]. In addition, although in this work the focus was on the preparation of plate-shaped PLA carriers, due to the user-friendly properties of 3D printing, any desired shape can be produced and functionalized in the same manner.

The NPs were tested for antibacterial activity by the agar well diffusion, as well as by the microdilution method against the Gram-positive bacterial model *S. aureus*. In order to assess the effects of colloidal metal and metal oxide nanoparticles, nine different model colloidal nanoparticle systems were investigated: Ag 40 nm (20 ppm), Ag 10 nm (20 ppm), Pt 4 nm (250, 225, and 100 ppm), ZnO 100 nm (10% and 21%), Au 20 nm (20 ppm), TiO_2_ 100 nm (33%), Al_2_O_3_ 100 nm (20%), Y_2_O_3_ 100 nm (5% and 10%) and ZrO_2_ 100 nm, (5%) by agar well diffusion and serial two-fold microdilution assays. Performed experiments included the standard laboratory Gram-positive bacterial strain from the American Type Culture Collection (ATCC, from the stock-cultures of the Collection of Microorganisms of the Department of Microbiology, Faculty of Pharmacy and Biochemistry, University of Zagreb, Croatia), i.e., *S. aureus* ATCC 29213, and a clinical isolate of MRSA from the stock-cultures of the Collection of Microorganisms of the Department of Microbiology, Faculty of Pharmacy and Biochemistry, University of Zagreb (MFBF, namely MRSA MFBF 10679). All microbial media were purchased from Merck (Darmstadt, Germany) and gentamicin sulphate was obtained from Sigma-Aldrich (St. Luis, MO, USA).

## 2. Materials and Methods

### 2.1. Chromatographic Characterization

**Figure 1:**
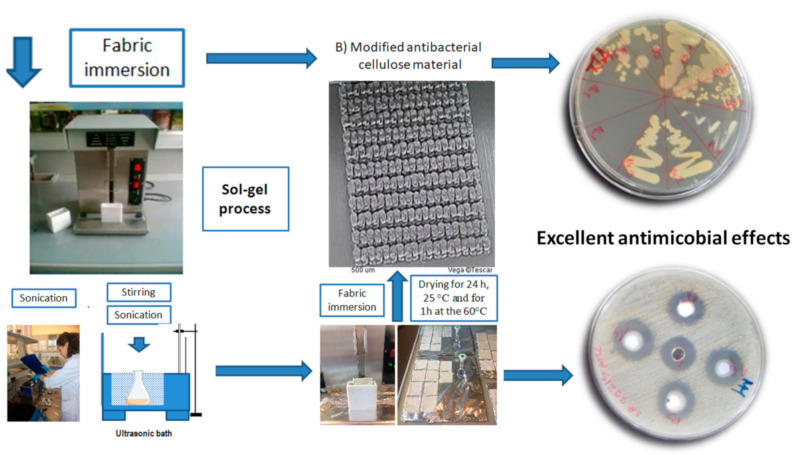
Schematic overview of different steps in preparation of the antimicrobially functionalized biodegradable polymers by sol–gel process and the resulting antimicrobial efficiency.

The main goal of the proposed research was to develop biodegradable antimicrobial polymers and create a method for characterization of metal nanoparticles (Figure 1). For this purpose, firstly the thin layer chromatography was tested for the analysis of 60 different elements, mostly metal ions (Ag, Al, As, Au, B, Ba, Be, Bi, Br, Ca, Cd, Co, Cr, Cu, Cs, Dy, Eu, Fe, Ga, Gd, Ge, Hf, Hg, Ho, In, Ir, K, Li, Lu, M, Mg, Mo, Na, Nb, Nd, Ni, Os, P, Pb, Pd, Pt, Rb, Re, Rh, Ru, S, Sb, Sc, Se, Si, Sn, Sr, Ta, Te, Th, Ti, Tl, U, V, W). All investigated elements can be produced in a form of metal or metal oxide nanoparticles with potential antimicrobial effects, if coated on biodegradable polymers. Secondly, the antimicrobial effects of up to nine different nanoparticles (metal and metalo-oxides) were tested (Table 1 and Table 2). The TLC method was developed for the analysis of metal ions that can be extracted from the functionalized polymers by metal and metalo oxide nanoparticles. Such nanoparticles can arise from the antimicrobial coating of biodegradable polymer samples. During preliminary experiments, 31 different stationary phases were tested (composed of thin layers of alumina oxide, cellulose, polyamide and silica gel). All of those stationary phases were eluted with different organic solvents and solvent mixtures which were investigated as mobile phases (acetonitrile and acetyl acetone, compared with butanol and triethylamine, or ethylenediaminotetraacetic acid). Moreover, inorganic compounds such as hydrochloric acid and water were added to particular mobile phases in order to create optimal solvent mixtures. In total, nineteen solvent mixtures were created during preliminary experimentation.

**Figure 2:**
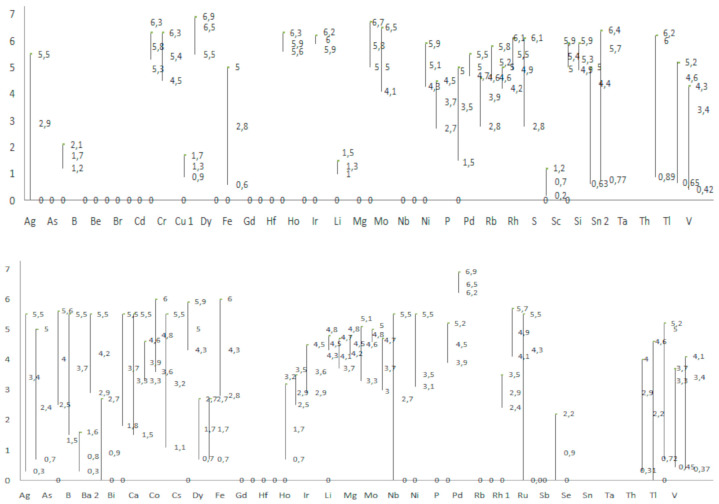
Graphical presentation of distribution of elements determined by stationary phase silicagel F_256_ (**above**) and cellulose F (**below**).

**Figure 3:**
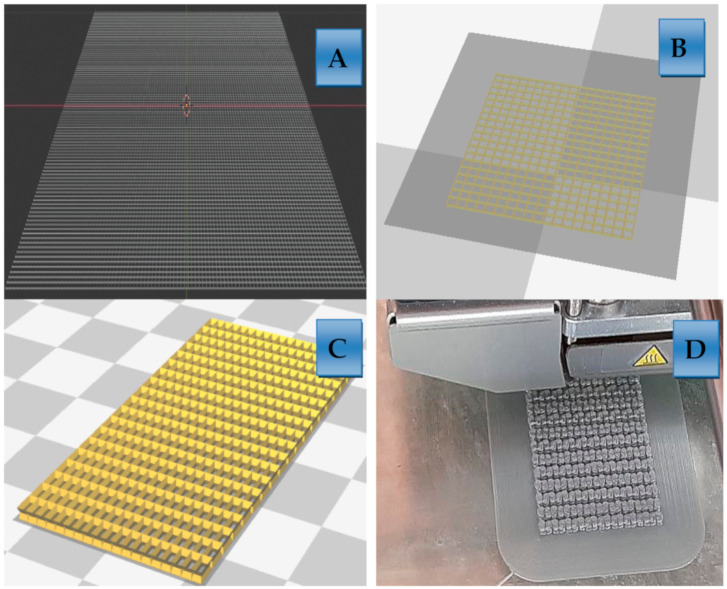
(**A**) Programming in Blender; (**B)** Model generating in the Cura; (**C**) converting model to slices and (**D**) 3D printing.

Finally, the optimized chromatographic method that was applied for separation and identification of all 60 metal ions contained silica gel as stationary phase, and mobile phase with three different solvents. Those solvents were namely ACN, HCl and H_2_O. Their volume ratio that was calculated after the optimization contained following volume rations: 60.00% of CAN, 19.17% of HCl and 20.83% of water. This optimal system was used and the collected results are shown in Figure 2 and Figure 3.

### 2.2. Preparation of Biodegradable Polymers as Carriers for Antimicrobial Coating

**Figure 4:**
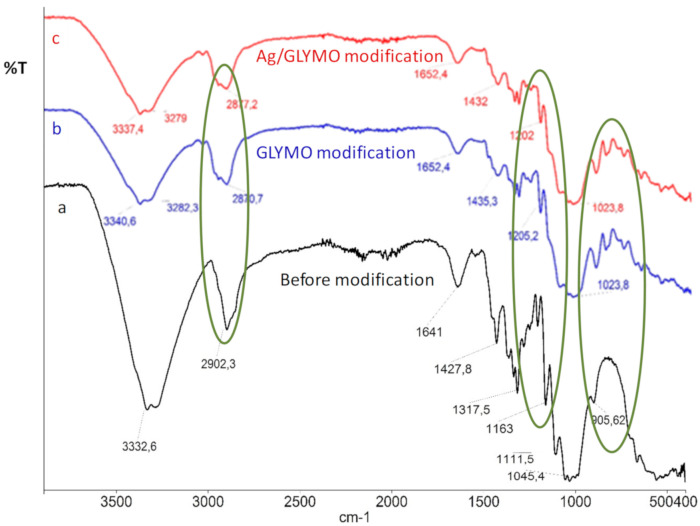
Fourier-transform infrared spectra of sample before and after the modification with the silver nanoparticles.

The second step in producing the antimicrobial polymers was to 3D print the desired surface that can be functionalized with an antimicrobial coating and afterwards be used as a disposable antibacterial item. The results are shown in Figure 1 and Figure 4. Such disposable items have applications in medicine and medical materials. Modern and sophisticated methodologies for producing small biodegradable materials are finding broad application in everyday life. Additive technology (3D printing) enables designing shapes that are printed after modeling. Such materials are foreseen to be used as catheters, consumables, dressings and wound dressings. With further modification, it would be possible to apply them in the production of protective masks and protective suits which are today some of the most frequent medical items on the global market.

The detailed description of the surface modification by using sol-gel procedure was described in our previous work [47]. In this previous work we have shown the methodology of polymer surface modification with ZnO nanoparticles. The process consists of dip-coating with precursor 3-glycidyloxypropyltrimethoxysilane (GLYMO, Sigma Aldrich) and HCl as a catalyst. Nanoparticles in sol state were left to gel at room temperature and then dried for 24 h at room temperature prior heating at the 100 °C for 60 min. Such modified products characterized by different instrumental techniques, including the thin layer chromatography (TLC), scanning electron microscopy with an EDX detector (SEM-EDX, Tescan Vega, Brno, Czech Republic) and FTIR-ATR (Perkin Elmer, MA, USA) spectroscopy [47]. In this work the results of modification by silver nanoparticles will be shown.

**Figure 5:**
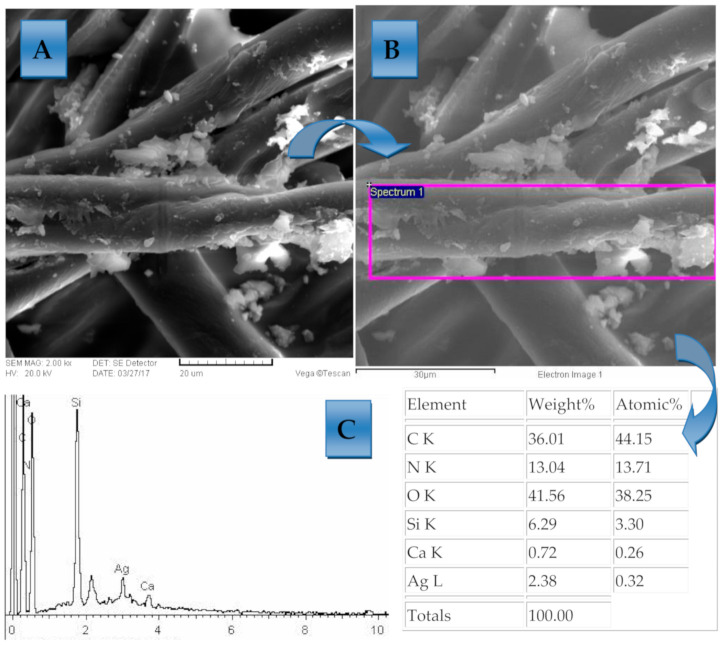
(**A**) SEM microphotograph of AgNPs and homogenous coating layer on polymers ma-terials with specified area used for EDX investigation; (**B**) The purple square is the area that was scanned for mapping; (**C**) Results of the SEM-EDX investigation with the proof that the cellulose material was successfully modified with the Ag coating. Magnification of (**C**) was 1000 times.

The samples after coating were tested using scanning electronic microscopy (TS5136LS” equipped with an EDS detector, Tescan Vega Co., Brno, Czech Republic). A microphotograph obtained from SEM microphotograph of the modified antimicrobial cellulose material, as well as EXD analysis of the sample, are presented in Figure 5 in the Results section.

### 2.3. Spectroscopical and Microscopical Characterization

For the purpose of the spectroscopic and microscopic characterization the following instrumentation was used: Fourier transform infrared spectrometer (Spectrum 100 FTIR, Perkin Elmer, Waltham, MA, USA), which applies the KBr and attenuated total reflectance (ATR) techniques and enables the recording of spectra of cellulose materials in their solid state. The spectra of samples were recorded in frequency range from 400 to 4000 cm^−1^ in diffuse reflectance mode, at a resolution of 4 cm^−1^.

### 2.4. Antimicrobial Effects against Stafilococus Aureus, Including MRSA and MSSA

For the purpose of this investigation, the antimicrobial activity investigation was performed on *S. aureus* model microorganism in two steps: firstly the agar well diffusion assay, and secondly by serial microdilution broth assay. The agar well diffusion assay was performed according to European Pharmacopoeia [32]. Briefly, inocula were prepared from fresh overnight bacterial cultures in physiological saline and optical density was adjusted to 0.5 McFarland units (Instrumentation: Kisker densitometer, Steinfurt, Germany). After inoculation, sample solutions were applied to wells in volumes of 50 µL. The pre-incubation of plates (that was performed at +4 °C for 1 h) was followed by incubation at the temperature of +37 °C for 18 h under aerobic conditions in dark.

Finally, the antimicrobial activity was evaluated by measuring the diameters of zones of growth inhibition (Z.I.; d, mm) around wells. Gentamicin sulphate (10 µg/mL) was used for quality control of the method and strain susceptibility. All tests were performed in quintuplicate and results were expressed as the mean ± SD, and the results are presented in Table 1.

In the second set of antimicrobial activity investigation, a serial microdilution broth assay was applied. In this investigation the minimal inhibitory concentrations (MICs) were determined for MSSA and MRSA strains. Minimal inhibitory concentration (MIC) was defined as the lowest concentration of metal and metalo-oxide nanoparticles which allowed 20% or less of microbial growth in comparison to the negative control. All tests were performed in triplicate and results were expressed as mean values ± SD.

Minimal inhibitory concentrations were guided by the serial microdilution broth assay, as specified in the guidelines of *The European Committee on Antimicrobial Susceptibility Testing (EUCAST E.Def.5.1)* [33]. As described in our previous work, the major modification was performed by replacing the medium Mueller–Hinton broth with physiological saline [42,47]. This step was performed in order to avoid the interfering activity of nanosized colloidal metal and metal oxide particles with the diverse protein contents present in the broth [42,47].

The investigation followed standard procedure of serial two-fold micro-dilution and incubation at 37 °C (with the time of incubation of 18–24 h) [33]. The standard reference materials of nanoparticles in their colloidal form were previously applied in serial and two-fold dilution sequences. After incubation, subcultivation of each concentration was performed by transferring 10 µL from each dilution well of sample on the surface of tryptic soy agar. The following procedure included re-incubation at the same temperature for 18 h. This step was performed in order to evaluate bacterial viability.

## 3. Results

### 3.1. The Results of Chromatographic Characterization

The main goal of the first part of this work was to develop and apply the thin layer chromatography method in the analysis of 60 different metals that can be potentially used, in a form of nanoparticles, in the antimicrobial coating of biodegradable polymer samples. After this step, the biodegradable polymer surface intended for functionalization with antimicrobial coating were designed, modeled and 3D printed prior functionalization with nanoparticles.

The chromatographic results of investigation cover data collected from 31 different types of stationary phases (including cellulose, silica gel, polyamide and alumina) which were tested, as well as numerous mobile phases consisting of various developers of mixtures of inorganic and organic solvents (acetonitrile, acetyl acetone, butanol, triethylamine, ethylenediaminetetraacetic acid). The optimal system by which metal ions were detected consisted of silica gel as a stationary phase, and acetonitrile: hydrochloric acid: water (60.00: 19.17: 20.83 v.v.). Optimization of this work was performed in 31 preliminary experiments. The optimal system was used to test all metals, and the results are presented in the next paragraph. Figure 2 shows the graphical presentations of the distribution of metals separated and determined by stationary phases cellulose and silica gel.

### 3.2. The Results of Preparation of Biodegradable Polymers as Carriers for Antimicrobial Coating

Biodegradable PLA materials were designed and prepared for 3D printing in several steps which are presented in Figure 3. This figure shows the procedure of preparation of biodegradable polymer carriers that are prepared to be coated with antibacterial active nanoparticles [40,41,42,43,44,45,46,47,48]. Such materials can also be used and produced in other shapes which are convenient to be catheters, consumables, or any other desired material needed in hospitals.

The first step of producing PLA carrier is the modeling of the biodegradable material before 3D printing and coating with nanoparticles. Firstly, the net was made in the *Blender* computer programme (Blender Institute, Amsterdam, The Netherland). The second step was modeling of the biodegradable material before 3D printing and coating with nanoparticles which was generated by the *Cura* programme (Ultimaker B.V., Utrecht, The Netherland). Thirdly, converting of the model into slices before 3D printing and coating with nanoparticles was preformed by the *Cura* program. The last step was final fussion deposition of melted biopolymer according to the prepraed model before coating with nanoparticles.

### 3.3. The Results of Spectroscopical and Microscopical Characterization

For the spectroscopic characterization of the biodegradable polymers, the Fourier-transform infrared spectrometry (Spectrum 100 FTIR) was applied as an efficient and reliable methodology for monitoring the presence of different functional groups on the polymer surface (Figure 4). This technique was chosen due to its excellent property to detect differences in newly formed functional groups on the investigated solid samples [47]. For this part of the research, the results of biodegradable cellulose polymers modified with the Ag NPs were used. The samples were afterwards observed by microscopically characterization using the SEM-EDX under 80× to 100× magnification (Figure 5). All nanoparticles used in this research work were in the form of colloidally stable solutions or suspensions. The nanoparticles were purchased with certified chemical composition and certified concentrations, as is shown in Table 1, Table 2 and Table 3.

As can be seen from Figure 4, the lines of samples before and after the sol–gel modification had distinguished differences. The black line in Figure 4 presented as the bottom line of the FTIR-attenuated total reflectance (ATR) spectra is the spectra of the sample before modification. Secondly, the middle blue line presents the FTIR ATR spectra of the sample after modification only with organic precursor reagents, namely with 3-glycidyloxypropyltrimethoxysilane (GLYMO). Thirdly, the upper red line is showing the FTIR ATR spectra of the sample modified both with Ag NPs and with the GLYMO precursor. Moreover, the three green circles define area of the functional groups before and after the modification: therefore the epoxy groups show peaks that can be determined around ~905 and 911 cm^−1^. In addition, the peaks around the area of 1100 cm^−1^ are linked to the Si–O groups, including the Si–O–C and Si–O–Si bridges. Moreover, the conversion of metoxy groups distinguished at ~2870 cm^−1^ were related to the precursor 3-glycidyloxypropyltrimethoxysilane.

The results of microphotographical characterization using the SEM-EDX technique showed that surfaces of sample materials were free of impurities, as well as proved the efficiency of the modification by proving the presence of the AgNPs. Figure 5 shows an SEM microphotograph of the surface with nanoparticle modification and Figure 5b shows the EDX spectra of this surface. As can be seen from these results, the result of the dip coating process was a uniform homogenous coating with coating of AgNPs.

### 3.4. The Results of Antimicrobial Testing

The results of agar well diffusion assay–antimicrobial investigation of metal and metalo-oxide nanoparticles on model microorganisms *S. aureus* ATCC 29213 are shown in Table 1. Table 2 and Table 3 present the obtained results of antimicrobial activity of colloidal metal and metalo-oxide nanoparticles on model microorganisms *S. aureus* ATCC 29213 (MSSA) and MRSA MFBF 10679 by the serial microdilution broth assay. Gentamicin sulphate was used as positive control, and pure physiological saline was used as the negative control.

## 4. Discussion

Preliminary evaluation included the optimization of chromatographic system for examination of the plates, as well as determination of the most suitable mobile phase. The importance of the preliminary test was in the selection of chromatographic plates that showed the best results of separation of metal ions, i.e., plates that can be used in further tests. The results of chromatographic plates with metal ions of silver, aluminum, arsenic, gold, bismuth, cadmium, cobalt, chromium, copper, iron, mercury, manganese, nickel, lead, antimony, silicon, tin and zinc are shown in Figure 2. During the test, a mixture of developers ACN, HCl, H_2_O, ratio 72: 25: 23 was used. The best separation results were shown by the plates *Polygram Cel 300, Silica gel, Cellulose F and hydrophobic plates HPTLC RP18 F_254_, HPTLC and HPTLC RP18;* while as for the hydrophobic plates, all of them showed similar results.

After 3D printing (Figure 3) relatively low resolution of carriers was achieved, and this was conditioned by the printer itself. Therefore, for medical application related to medical implants, much more optimization of polymers and parameters of fusion needs to be done. Therefore, the ultra fine network structure with extra high resolution cannot be obtained by using an Ultimaker 2+ system (Ultimaker B.V., Utrecht, The Netherland). The surface of such tiles was uneven and therefore rough, while the cross section had the shape of a “sandwich”. Nevertheless, despite the low resolution, thus printed plates were ready for further processing by sol-gel process for the purpose of functionalization of biodegradable polymers into antimicrobial materials. This research is the extension of our previous results in which we have managed to obtain antimicrobial surface [42] and very hydrophobic materials [43,47].

In this work, as can be seen from the reference 42, the antibacterial effect of our antimicrobial polymer coated with nanoparticles started after 6 h of exposure [42]. All viable bacteria were eliminated after 24 h for all investigated samples, except for sample code 11 in the case of MSSA, where no bacterial growth was observed after 18 h. As can be seen from the results presented in the Table 1, among all tested samples and available maximal colloidal concentrations, the antimicrobial activity was observed as follows: highest values of Z.I. were measured for ZnO samples (100 nm), followed by Pt (4 nm) and Ag (10 nm and 40 nm) (Table 2). The concentrations used for the research were chosen based on the parameters of the maximal colloidal stability of the nanoparticles. All nanoparticles used in this investigation were colloidal stabile solutions with nanoparticles of metals and metal oxides differing in size and chemical composition. Due to these parameters, also their maximal stabile concentrations differed which can be calculated according to the Derjaguin–Landau–Verwey–Overbeek (DLVO) theory [48]. According to this theory, the stability phenomenon is explained by using the attractive van der Waals forces and the repulsive forces of the electric double layer. The most important parameter for increasing the stability of the colloid was the increased net surface charge values. Therefore, for the colloid solutions containing nanoparticles that have amphoteric surfaces, such are metal oxides, their electric double layer was strongly influenced by the ionic strength of the colloid. In conclusion, different colloid systems containing metal and metal oxide nanoparticles had different maximal concentrations that determined their stability. If higher concentrations would have been used, this would have resulted with the compression of the double layer and finally with the collapse of the stability of the whole colloidal nanoparticles system [48]. Maximal concentrations of colloid metal oxide nanoparticles were in range from minimal 5% (for ZrO_2_), 10% (Y_2_O_3_), 20% and 21% (for Al_2_O_3_ and ZnO) up to maximal 33% (for TiO_2_).

Moreover, the results of the antimicrobial investigation of metal and metalo oxide nanoparticles on model microorganism *S. aureus* ATCC 29213 by the agar well diffusion assay presented in the Table 1 for ZnO particles (100 nm) have shown that the antimicrobial effects depended strongly on the concentration of the nanoparticle: the concentration of 21% of ZnO nanoparticles resulted with the 37 ± 2 mm zone of inhibition, while in contrast 10.5% solution of ZnO nanoparticles obtained 15 ± 2 mm. By this it was shown that the antimicrobial efficiency depended not only on the chemical composition of nanoparticles and their size, but also on the concentration of the solution used for the treatment.

ZnO (100 nm) showed the highest antimicrobial activity on MSSA, with MBC value 0.14 ± 0.06 ppm and MIC value 0.03 ± 0.00 ppm [42,47]. On the contrary, TiO_2_ (100 nm) was least active with MBC 80.57 ± 0.00 ppm and MIC 40.28 ± 0.00 ppm (Table 2). The most active among investigated samples in regards of activity against MRSA was Ag (10 nm) with MBC 2.50 ± 0.00 ppm and MIC 0.47 ± 0.27 ppm, while the least active sample was TiO_2_ (100 nm) (Table 3).

When comparing the results of the antimicrobial activity on MSSA presented in Table 2, it can be concluded that the MBC values were higher than the MIC values for all investigated colloid nanoparticles. For example, MBC values of Pt (4 nm) was 0.20 and MIC 0.06; while for Ag NPs MBC was 1.04, and MIC 0.02. A similar trend was observed for TiO_2_ NPs where MBC and MIC values of 80.57 and 40.28, respectively, were measured. The differences between MIC and MBC values rise in a following order: MBC of the TiO_2_ (100 nm) is 2 times higher than its MIC value, for Pt (4 nm) and Ag (10 nm) is 3.3 and 3.4 times higher, for ZnO (100 nm) is 4.6 times higher. In some cases the differences are not very high (and are in ranges from 2 to 4.6 times for Pt (4 nm) and Ag (10 nm), respectively). In contrary, the results for Ag (40 nm), and ZrO (100 nm) differences are much more prominent: 52 times for Ag (40 nm) and 208 times for ZrO_2_ (100 nm). Moreover, it is interesting to observe the influence of the silver nanoparticles that are different in size: while differences between MBC and MIC values for Ag (10 nm) show only 3.4 higher values, the results for Ag (40 nm) are 52 times higher. Observed MBC of Ag (10 nm) is lower (0.31 ± 0.27 ppm) than for Ag (40 nm) (1.04 ± 1.26 ppm). This observed difference is in agreement with previous literature data, where smaller nano sized Ag shows higher antimicrobial activity [49]. The more active Ag (10 nm) was then also tested on MRSA and showed somewhat lower, but very similar activity as on MSSA (Table 3).

Table 3 presents the results of the antimicrobial activity on MRSA model microorganisms obtained for three selected nanoparticles: Ag (10 nm), ZnO (100 nm) and TiO_2_ (100 nm). Those colloid systems were chosen due to the fact that silver, ZnO and TiO_2_ are the most widely used antimicrobial reagents on the market. Since this research aims to develop a possible medical material, we have chosen the approach to use nanoparticles that are already in use on the market for other microbial effects, and to test them on MRSA model microorganisms. Moreover, from the Table 3 it can be concluded that the MIC values with Ag (10 nm) nanoparticles were 5.2 times higher for MRSA than for MSSA, and 109.3 times higher with ZnO (100 nm). It is known from the literature data that mechanism of MRSA antibiotic resistance isn’t applying on nano sized ZnO, but in this research a statistically valid difference in MIC and MBC values was observed in comparison to the MSSA strain: it was higher for MRSA with MIC 3.28 ± 0.55 ppm and MBC 3.28 ± 1.89 ppm [50]. In contrary, for TiO_2_ (100 nm) nanoparticles MIC value for MSSA was 2 times higher than for the MRSA.

Similarity in behavior was observed when comparing the MBC values which were in all cases higher for MRSA than for MSSA strains. Namely, those were 23.43 times higher for MRSA than MSSA by using the ZnO (100 nm) nanoparticles, 8.07 times higher with Ag (10 nm) nanoparticles, and only 0.1 times higher with TiO_2_ (100 nm) nanoparticles.

Application of antimicrobial active colloidal nanoparticles efficient against MSSA and MRSA was the main focus of this work. The efficiency of the newly formed chemical bonds as a result of functionalization is shown at the FTIR-ATR spectra of samples before an after modification. As can be seen from the Figure 4, new chemical bonds occurred which proves the efficiency of the sol-gel methodology. The peaks around the area of 1100 cm^−1^ were linked to the Si–O groups, and the conversion of the precursor 3-glycidyloxypropyltrimethoxysilane’s metoxy groups were distinguished at ~2870 cm^−1^. Therefore the antibacterial coating was efficiently applied on the polymer surface. The antibacterial activity and mechanism of Ag NPs on *S. aureus* strain ATCC 6538P was also investigated by Li and coauthors [44]. Their results have shown that the minimal bactericidal concentration (MBC) is 20 μg/mL. When compared to the results of Mirzajani and coworkers [45], who investigated the antibacterial activity of silver nanoparticles against S*. aureus* strain PTCC1431, the data showed that AgNPs at a concentration of 4 μg/mL completely inhibited bacterial growth. Moreover, when *S. aureus* ATCC 6538P was exposed to silver nanoparticles of a higher concentration, namely 50 μg/mL in period of 6 h, the cell DNA was affected and after 12 h, the cell wall broke. The release of the cellular contents into the surrounding environments was observed, with the final result of the total cell collapse [44]. This interaction was confirmed by Grigoreva et al. [46], who proved direct interaction between silver nanoparticles and *S. aureus* strain’s macromolecular structures. The results of our research confirmed the literature data. We have shown that the Ag NPs and ZnO NPs are the most prominent candidates for application on the antibacterial medical materials produced from biodegradable polymers.

## 5. Conclusions

The results of this interdisciplinary study have shown that there are many different issues to be addressed in finding optimal nano-technological medical solutions to deal with bacterial infections. Due to the COVID-19 pandemic, and the huge increase in the number of hospitalized patients, demands for finding antimicrobial materials are rising. This study aimed to answer several the most prominent questions regarding functionalization and preparation of antimicrobial coatings active against *S. aureus* infections or colonization, including MSSA and MRSA strains. It was shown that many metal and metalo-oxide nanoparticles show antimicrobial effects in vitro, and that by 3D printing any desired polymer carrier shape can be prepared for medical purposes. The most prominent colloidal nanoparticles for medical materials are colloid silver particles (size 10 nm), and zinc oxide nanoparticles (in size of 100 nm). In addition, an attempt to develop a chromatographic methodology for simultaneous separation and determination of trace quantities of analyte was presented. However, this is just the first step and our future investigation will need to include further research results on the activity of particular nanoparticles, such as for example Pt (4 nm).

## Figures and Tables

**Table 1:** Results of antimicrobial investigation of metal and metalo oxide nanoparticles on model microorganism *S. aureus* ATCC 29213 by agar well diffusion assay (n = 5).

Nanoparticles	Size (nm)	Concentration *	Z. I./mm (Mean ± SD)
**Pt**	4	225 ppm	12 ± 2
		250 ppm	11 ± 2
		12.5–100 ppm	0 ± 0
**Ag**	10	20 ppm	10 ± 1
	40	20 ppm	10 ± 1
**ZnO**	100	21%	37 ± 2 [42,47]
		10.5%	15 ± 2 [42,47]
**TiO_2_**	100	33%	0 ± 0
**Al_2_O_3_**	100	20%	0 ± 0
**Y_2_O_3_**	100	10%	0 ± 0
		5%	0 ± 0
**ZrO_2_**	100	5%	0 ± 0
**Gentamicin suphate (positive control)**		10.0 µg/mL	18 ± 2

* 1% = 10,000 ppm; Z. I.—zone of growth inhibition.

**Table 2:** Results of antimicrobial activity investigation of metal and metalo oxide nanoparticles on model microorganism *S. aureus* ATCC 29213 (MSSA) by serial microdilution broth assay (MIC—minimal inhibitory concentration; MBC—minimal bactericidal concentration; n = 3).

Nanoparticle (Size)	MIC (ppm)	MBC (ppm)
	Mean ± SD	
Pt (4 nm)	0.06 ± 0.04	0.20 ± 0.17
Ag (10 nm)	0.09 ± 0.07	0.31 ± 0.27
Ag (40 nm)	0.02 ± 0.02	1.04 ± 1.26
ZnO (100 nm)	0.03 ± 0.00 [42,47]	0.14 ± 0.06 [42,47]
TiO_2_ (100 nm)	40.28 ± 0.00	80.57 ± 0.00
Y_2_O_3_ (100 nm)	<12.21	12.21 ± 0.00
ZrO_2_ (100 nm)	<0.06	>12,500.00
Gentamicin sulphate (positive control)		0.63 ± 0.00 µg/mL

**Table 3:** Results of antimicrobial activity investigation of metal and metalo oxide nanoparticles on model microorganism MRSA MFBF 10679 by serial micro dilution broth assay (MIC—minimal inhibitory concentration; MBC—minimal bactericidal concentration; n = 3).

Nanoparticle (Size)	MIC (ppm)	MBC (ppm)
	Mean ± SD	
Ag (10 nm)	0.47 ± 0.27	2.50 ± 0.00
ZnO (100 nm)	3.28 ± 0.55	3.28 ± 1.89
TiO_2_ (100 nm)	20.14 ± 0.00	93.00 ± 61.53
Gentamicin sulphate (positive control)		1.00 ± 0.40 µg/mL

## Data Availability

Not applicable.
